# Time course of the effects of lisdexamfetamine dimesylate in two phase 3, randomized, double‐blind, placebo‐controlled trials in adults with binge‐eating disorder

**DOI:** 10.1002/eat.22722

**Published:** 2017-05-08

**Authors:** Susan L. McElroy, James I. Hudson, Maria Gasior, Barry K. Herman, Jana Radewonuk, Denise Wilfley, Joan Busner

**Affiliations:** ^1^ Lindner Center of HOPE Mason Ohio; ^2^ Department of Psychiatry and Behavioral Neuroscience University of Cincinnati College of Medicine Cincinnati Ohio; ^3^ Department of Psychiatry McLean Hospital/Harvard Medical School Belmont Massachusetts; ^4^ Formerly of Shire Lexington Massachusetts; ^5^ Department of Psychiatry Washington University School of Medicine St Louis Missouri; ^6^ Department of Psychiatry Penn State College of Medicine Hershey Pennsylvania; ^7^ Bracket Wayne Pennsylvania

**Keywords:** binge‐eating disorder, efficacy, lisdexamfetamine, time course

## Abstract

**Objective:**

This study examined the time course of efficacy‐related endpoints for lisdexamfetamine dimesylate (LDX) versus placebo in adults with protocol‐defined moderate to severe binge‐eating disorder (BED).

**Methods:**

In two 12‐week, double‐blind, placebo‐controlled studies, adults meeting *DSM‐IV‐TR* BED criteria were randomized 1:1 to receive placebo or dose‐optimized LDX (50 or 70 mg). Analyses across visits used mixed‐effects models for repeated measures (binge eating days/week, binge eating episodes/week, Yale‐Brown Obsessive Compulsive Scale modified for Binge Eating [Y‐BOCS‐BE] scores, percentage body weight change) and chi‐square tests (Clinical Global Impressions—Improvement [CGI‐I; from the perspective of BED symptoms] scale dichotomized as improved or not improved). These analyses were not part of the prespecified testing strategy, so reported *p* values are nominal (unadjusted and descriptive only).

**Results:**

Least squares mean treatment differences for change from baseline in both studies favored LDX over placebo (all nominal *p* values <  .001) starting at Week 1 for binge eating days/week, binge‐eating episodes/week, and percentage weight change and at the first posttreatment assessment (Week 4) for Y‐BOCS‐BE total and domain scores. On the CGI‐I, more participants on LDX than placebo were categorized as improved starting at Week 1 in both studies (both nominal *p* values <  .001). Across these efficacy‐related endpoints, the superiority of LDX over placebo was maintained at each posttreatment assessment in both studies (all nominal *p* values <  .001).

**Discussion:**

In adults with BED, LDX treatment appeared to be associated with improvement on efficacy measures as early as 1 week, which was maintained throughout the 12‐week studies.

## 1 INTRODUCTION

Lisdexamfetamine dimesylate (LDX) is approved for use in adults with moderate to severe binge‐eating disorder (BED) in the United States (Vyvanse^®^, 2015). In two large, identically designed, 12‐week, dose‐optimized, randomized, double‐blind, placebo‐controlled, Phase 3 studies in adults with protocol‐defined moderate to severe BED, dose‐optimized LDX (50 or 70 mg/day) produced statistically superior and clinically meaningful reductions in binge eating days/week at weeks 11–12 (primary efficacy endpoint) versus placebo (McElroy et al., 2015a). In these Phase 3 trials, statistically significant and clinically meaningful improvements on secondary efficacy endpoints were also observed for LDX versus placebo at Week 12/early termination (ET) for including Clinical Global Impressions—Improvement (CGI‐I) and 4‐week binge eating cessation and at Week 12 for Yale‐Brown Obsessive Compulsive Scale modified for Binge Eating (Y‐BOCS‐BE) and percentage change in body weight (McElroy et al., 2015a). In a Phase 2, fixed‐dose, randomized, double‐blind, placebo‐controlled trial, LDX (50 and 70 mg but not 30 mg) was also superior to placebo in decreasing binge eating days/week in adults with BED at Week 11 (McElroy et al., 2015b). In these short‐term studies (McElroy et al., 2015a, 2015b), the safety and tolerability of LDX were generally similar to its established profile for LDX treatment of attention‐deficit/hyperactivity disorder (ADHD) (Vyvanse^®^, 2015).

Although the main findings for the primary and key secondary efficacy endpoints from the Phase 3 studies of LDX have been published (McElroy et al., 2015a), these analyses only examined change from baseline to end of study (Week 12 or Week 12/ET). Statistical assessment of the time course of effects of LDX on efficacy‐related endpoints from these studies has not yet been reported. Such data are important because they provide an indication of how soon treatment effects may be anticipated after the therapy is initiated. The current report describes the time course of effects of LDX on efficacy‐related endpoints (binge eating days/week, binge eating episodes/week, percentage of participants exhibiting improvement on the dichotomized CGI‐I, percentage of participants exhibiting 1‐week binge eating response, percentage change in body weight, and Y‐BOCS‐BE total and subscale score changes) in the two previously described 12‐week treatment Phase 3 clinical studies (McElroy et al., 2015a).

## 2 METHOD

### 2.1 Study design and treatment

Detailed descriptions of study designs and participants have been reported (McElroy et al., 2015a). In brief, two randomized, placebo‐controlled, parallel‐group, multicenter studies (ClinicalTrials.gov identifier: NCT01718483 [referred to hereafter as Study 1] and ClinicalTrials.gov identifier: NCT01718509 [referred to hereafter as Study 2]) were conducted. Study protocols were approved by ethics committees, and both studies were conducted in accordance with International Conference on Harmonisation Good Clinical Practice and the principles of the Declaration of Helsinki. Participants provided written informed consent before entering the studies.

Each study included a 2‐week screening phase, a 12‐week double‐blind phase (4 weeks of dose optimization followed by 8 weeks of dose maintenance), and a follow‐up visit. After screening, participants were randomized 1:1 to receive 12 weeks of dose‐optimized LDX or matching placebo. For blinding, both treatments were identical in appearance. Treatment was initiated with 30 mg LDX during Week 1 and titrated to 50 mg LDX at the start of Week 2. During Week 3, dose increases to 70 mg LDX could be made based on tolerability and clinical need. After the LDX dose was increased to 70 mg, a single down‐titration to 50 mg was allowed during Week 3 if tolerability to 70 mg LDX was poor. If a dose reduction occurred, no further changes were allowed. During Weeks 4 to 12, the optimized LDX dosage (50 or 70 mg) was maintained. Because no dose changes were permitted beyond Week 3, participants requiring a dose reduction during the maintenance phase were discontinued. A follow‐up visit occurred 1 week after the final treatment visit (Week 12 or ET) to assess any ongoing or new safety and tolerability issues.

### 2.2 Participants

Participant inclusion and exclusion criteria have been reported previously (McElroy et al., 2015a). Eligible adults (aged 18–55 years) met the *Diagnostic and Statistical Manual of Mental Disorders, Fourth Edition, Text Revision* (*DSM‐IV‐TR*) criteria for BED and had protocol‐defined moderate to severe BED (defined as having ≥3 binge eating days/week for 14 days before baseline and Clinical Global Impressions—Severity scores [from the perspective of binge eating symptoms] at screening and baseline of ≥4). Key exclusion criteria included current anorexia nervosa or bulimia nervosa; comorbid current psychiatric disorders either controlled with prohibited medications or uncontrolled and associated with significant symptoms or any condition that could confound study assessments; lifetime history of psychosis, mania, hypomania, dementia, or ADHD; a Montgomery–Åsberg Depression Rating Scale total score ≥18 at screening; psychotherapy or weight loss support for BED within 3 months of screening; being considered a suicide risk in the opinion of the investigator, having a previous suicide attempt, or currently demonstrating active suicidal ideation; history of cardiovascular disorders or moderate or severe hypertension; and lifetime history of stimulant abuse, recent history of substance abuse or dependence, or known or suspected intolerance or hypersensitivity to LDX or related compounds.

### 2.3 Endpoint measures

Binge eating days/week, binge eating episodes/week, and 1‐week binge eating response (percentage reductions in binge eating episodes/week) data were based on participants' daily self‐reported binge eating diaries as assessed and confirmed by experienced and trained clinicians. Binge eating diaries were assessed at all study visits except screening. The percentage of participants exhibiting 1‐week binge eating responses (reductions in binge‐eating episodes/week of 100%, 99–75%, 74–50%, and <50%) was derived at each treatment visit.

The CGI‐I (Guy, 1976) measured changes in clinical severity relative to baseline (score range: 1 [very much improved] to 7 [very much worse]). The CGI‐I was assessed from the perspective of BED symptoms and administered at each postbaseline visit. CGI‐I scores were dichotomized as improved (very much improved and much improved; scores of 1 or 2) or not improved (minimally improved to very much worse; scores of 3–7).

The Y‐BOCS‐BE, a 10‐item, clinician‐rated scale (item scores: 0 [no symptoms] to 4 [extreme symptoms]), assessed the obsessiveness of binge eating thoughts and compulsiveness of binge eating behaviors. Total scores range from 0 to 40. The Y‐BOCS‐BE, which is a modified version of the Yale‐Brown Obsessive Compulsive Scale (Goodman et al., 1989), has been validated in adults with BED (Deal et al., 2015). The Y‐BOCS‐BE was administered at baseline and Weeks 4, 8, and 12. Body weight was measured at each visit, and the percentage change from baseline was calculated for each treatment week.

### 2.4 Data presentation and statistical analyses

Statistically significant findings for the prespecified primary efficacy endpoint (change from baseline in binge eating days/week at Weeks 11–12) and key secondary endpoints (improvement on the dichotomized CGI‐I at Week 12/ET, 4‐week binge cessation at Week 12/ET, percentage body weight change from baseline at Week 12, and Y‐BOCS‐BE total score change from baseline at Week 12) have previously been reported (McElroy et al., 2015a). Time course assessments of efficacy‐related endpoints are described in this report.

Statistical assessments were conducted in the full analysis set (participants taking ≥1 study drug dose and having ≥1 postbaseline primary efficacy assessment). The time course analyses presented in the current report are analyzed with the same procedures previously described for the prespecified endpoints (McElroy et al., 2015a). Mixed‐effects models for repeated measures analysis over all postbaseline visits, using an unstructured covariance matrix with treatment, visit, and the treatment × visit interaction included as factors and baseline score as a covariate and its interaction with visit also included in the model, were used to determine least squares (LS) mean treatment differences (LDX—placebo) in the change from baseline for binge eating days/week, binge‐eating episodes/week, percentage body weight change, and Y‐BOCS‐BE total and domain scores; effects size (ES) size was determined based on the estimated standard deviation from the unstructured covariance matrix. Degrees of freedom were calculated using the Kenward‐Roger approximation method. Treatment comparisons between LDX and placebo in the percentage of participants categorized as improved on the dichotomized CGI‐I were analyzed using χ^2^ tests; odds ratios (ORs) were calculated as LDX/placebo. For 1‐week binge eating response (percentage reductions in binge eating episodes/week), differences in the distribution of responses between LDX and placebo were compared using a covariate‐adjusted Cochran–Mantel–Haenszel method, with baseline binge eating episodes/week included as the covariate. Cramer's V was calculated using a 2 × 4 contingency table at each visit to assess the association between the treatment group and binge eating response; values range from 0 (no association) to 1 (complete association).

Consistent with International Conference on Harmonisation statistical guidelines (European Medicines Agency, 1998), the control of multiplicity in each study was prespecified in the statistical analysis plan; these procedures have previously been described (McElroy et al., 2015a). The current post hoc time course analyses were not adjusted for multiple comparisons. As such, the *p* values reported for these post hoc time course analyses are nominal (unadjusted for multiplicity and descriptive). In the results, descriptions of the post hoc time course analyses and of the prespecified analyses included in the hierarchical testing strategy are presented separately to more clearly differentiate these analyses.

## 3 RESULTS

### 3.1 Participant disposition and demographics

Participant disposition has previously been reported (McElroy et al., 2015a). In brief, most randomized participants completed each study. A total of 68 participants did not complete Study 1 and 96 participants did not complete Study 2. Relatively few participants discontinued because of adverse events or lack of efficacy. The full analysis set included 374 participants in Study 1 and 350 participants in Study 2.

In Study 1 and Study 2, respectively, most participants in the full analysis set were white (77.8% [291/374] and 74.0% [259/350]), were women (86.9% [325/374] and 86.3% [302/350]), and met criteria for obesity (BMI ≥ 30.0 but <35 kg/m^2^: 25.4% [95/374] and 27.7% [97/350]; BMI ≥ 35.0 but <40 kg/m^2^: 24.1% [90/374] and 22.3% [78/350]; BMI ≥ 40.0 kg/m^2^: 17.6% [66/374] and 19.7% [69/350]). Mean ± *SD* age and BMI, respectively, were 38.0 ± 10.32 years and 33.43 ± 6.245 kg/m^2^ in Study 1 and 38.0 ± 10.04 years and 33.61 ± 6.272 kg/m^2^ in Study 2.

### 3.2 Efficacy analyses

#### 3.2.1 Binge eating days/week and binge‐eating episodes/week

**Figure 1:**
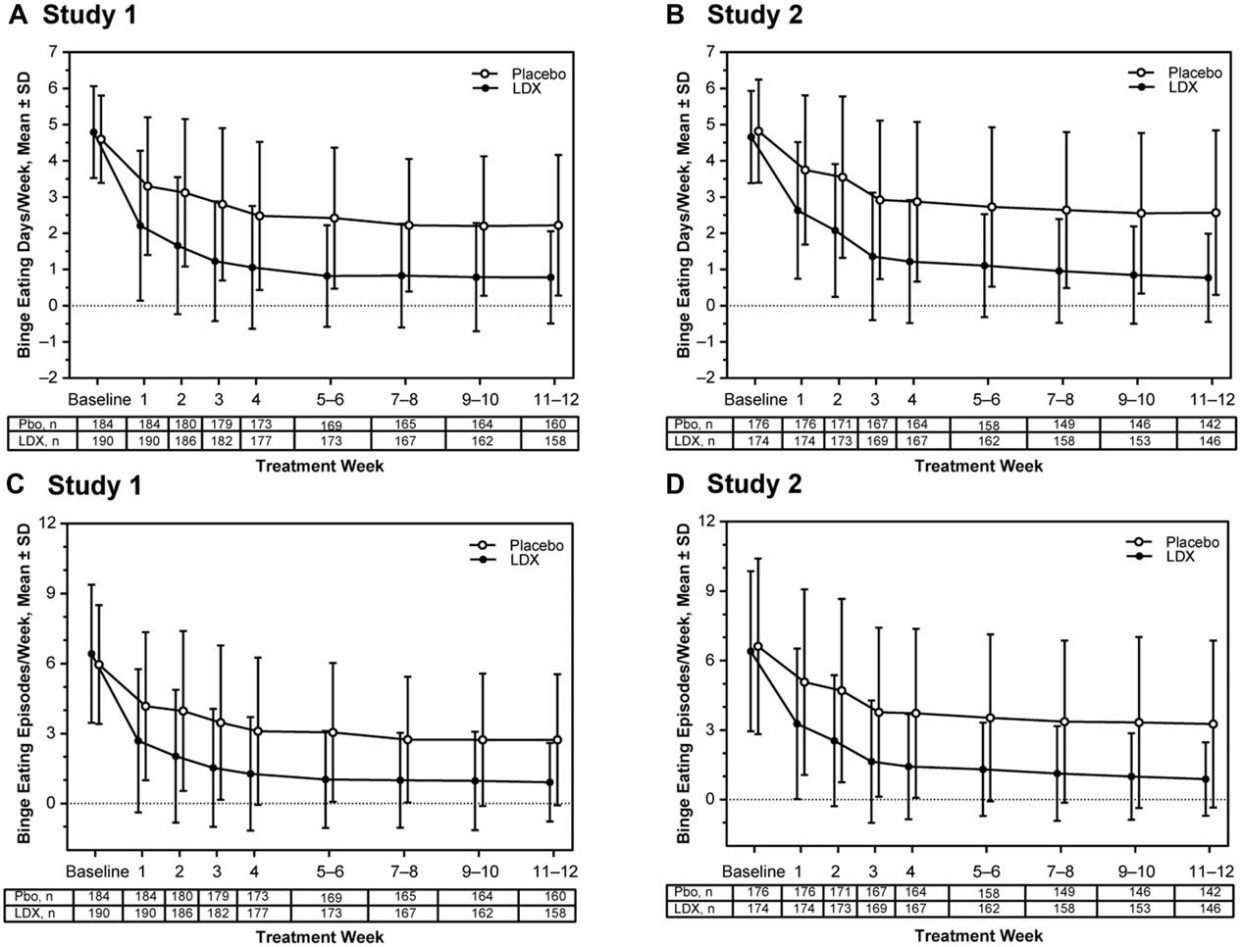
Changes in the frequency of binge eating over time, full analysis set. Mean ± *SD* binge eating days/week (A: Study 1; B: Study 2) and mean ± *SD* binge‐eating episodes/week (C: Study 1; D: Study 2). Abbreviations: LDX=lisdexamfetamine; Pbo=placebo

The baseline mean ± *SD* numbers of binge eating days/week and binge‐eating episodes/week, respectively, were 4.60 ± 1.210 and 5.96 ± 2.551 with placebo and 4.79 ± 1.271 and 6.42 ± 2.962 with LDX in Study 1 and 4.82 ± 1.422 and 6.62 ± 3.797 with placebo and 4.66 ± 1.273 and 6.40 ± 3.463 with LDX in Study 2. The mean ± *SD* numbers of binge eating days/week (Figure 1A,B) and binge eating episodes/week (Figure 1C,D) decreased with placebo and LDX from Week 1 through Weeks 11–12 in both studies. For binge eating days/week, LS mean (95% CI) treatment differences favored LDX from Week 1 through Weeks 9–10 (all nominal *p* values < .001; all ES ≥  .57) and at Weeks 11–12 (both *p* values <  .001; both ES ≥  .83) in both studies (Supporting Information Table 1). For binge eating episodes/week, LS mean (95% CI) treatment differences also favored LDX over placebo from Week 1 through Weeks 11–12 (all nominal *p* values < .001; all ES ≥ .60; Supporting Information Table 1).

##### 3.2.1.1 Improvement on the CGI‐I

**Figure 2:**
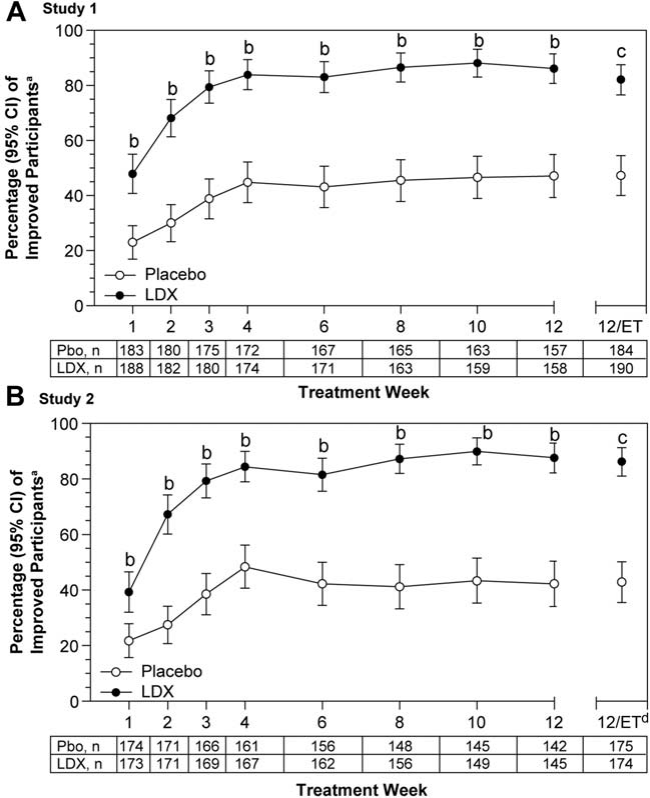
Improvement on the CGI‐I over time, full analysis set. Percentage (95% CI) of participants categorized as improved^a^ on the CGI‐I (A: Study 1; B: Study 2). ^a^Participants were categorized as improved on the CGI‐I if a score of 1 (very much improved) or 2 (much improved) was reported. ^b^Nominal *p* < .001; ^c^ *p* < .001 (prespecified key secondary endpoint included in the hierarchical testing strategy, data previously published [McElroy et al, 2015a]). ^d^One participant in the placebo group in Study 2 did not have a valid Week 12/early termination assessment. Abbreviations: CGI‐I = Binge Eating Clinical Global Impressions–Improvement; ET=early termination; LDX=lisdexamfetamine; Pbo=placebo

The percentage of participants categorized as improved on the CGI‐I increased over the course of both studies with placebo and LDX (Figure 2A,B). In both studies, the percentage of participants categorized as improved was greater with LDX than placebo from Week 1 through Week 12 (all χ^2^ statistics ≥ 12.48; degrees of freedom = 1; all nominal *p* values < .001; all ORs ≥ 2.32) and at Week 12/ET (both χ^2^ statistics ≥ 49.81; degrees of freedom = 1; both *p* values < .001; both ORs ≥ 5.12).

###### 3.2.1.1.1 1‐Week binge eating response (percentage reductions in binge‐eating episodes/week)

**Figure 3:**
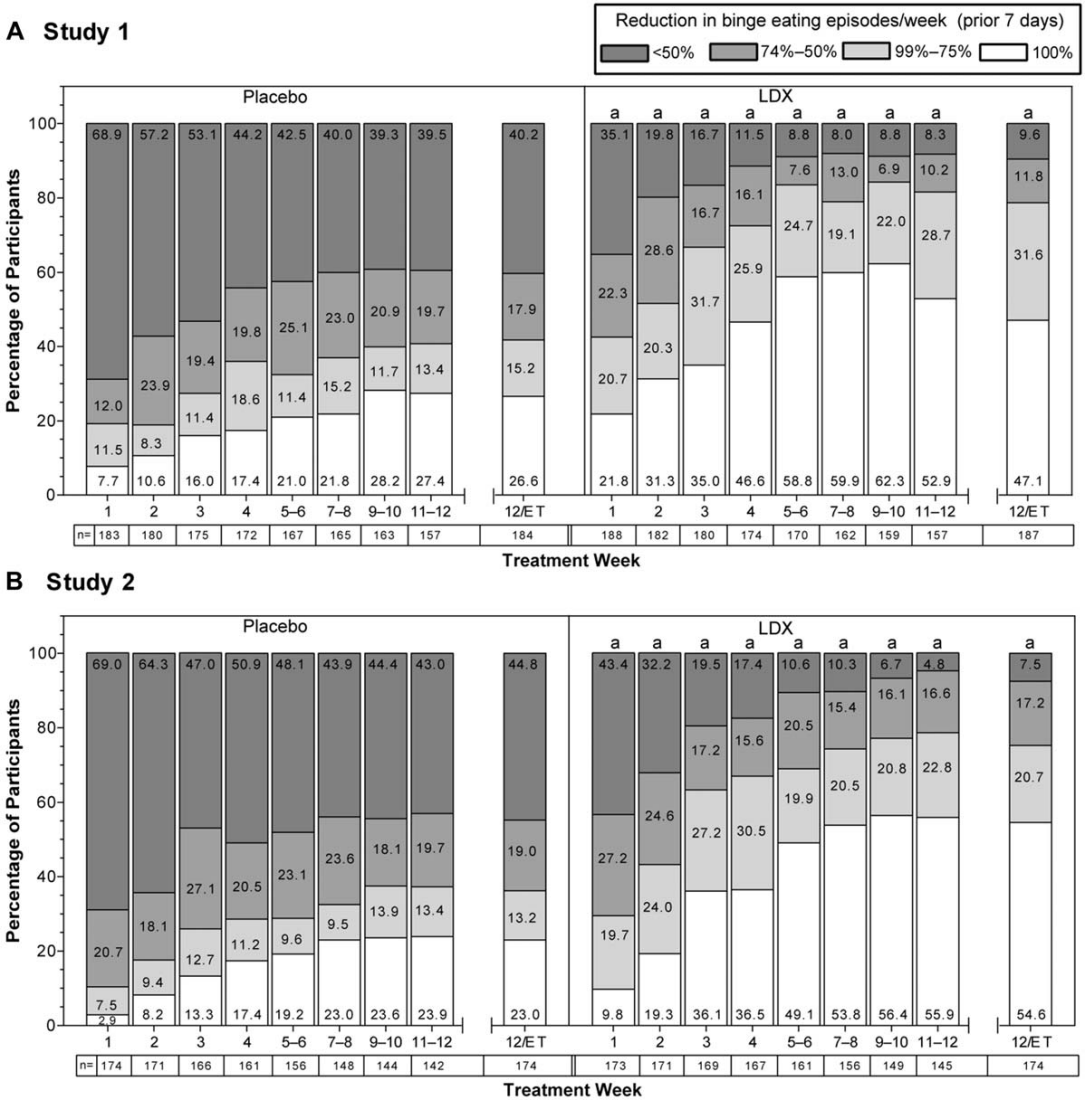
1‐week binge eating responses (reductions in binge‐eating episodes/week) over time, full analysis set. Percentages of participants exhibiting reductions in binge eating episodes/week over the prior 7 days (A: Study 1; B: Study 2). ^a^Nominal *p* < .001 for distribution of binge eating responses. Abbreviations: ET = early termination; LDX = lisdexamfetamine

The 1‐week binge eating response distributions differed between LDX and placebo in both studies from Week 1 through Week 12 (all χ^2^ statistics ≥ 16.66 based on Cochran–Mantel–Haenszel tests; degrees of freedom = 1; all nominal *p* values < .001; all Cramer's Vs ≥ .28) and at Week 12/ET (both χ^2^ statistics ≥ 43.82 based on Cochran–Mantel–Haenszel tests; degrees of freedom = 1; both nominal *p* values < .001; both Cramer's Vs ≥ .40). The percentages of participants exhibiting binge eating episode reductions of 100% and 99% to 75% in the last 7 days were numerically greater with LDX than placebo from Week 1 to Week 12 in both studies (Figure 3A,B).

#### 3.2.2 Body weight

**Figure 4:**
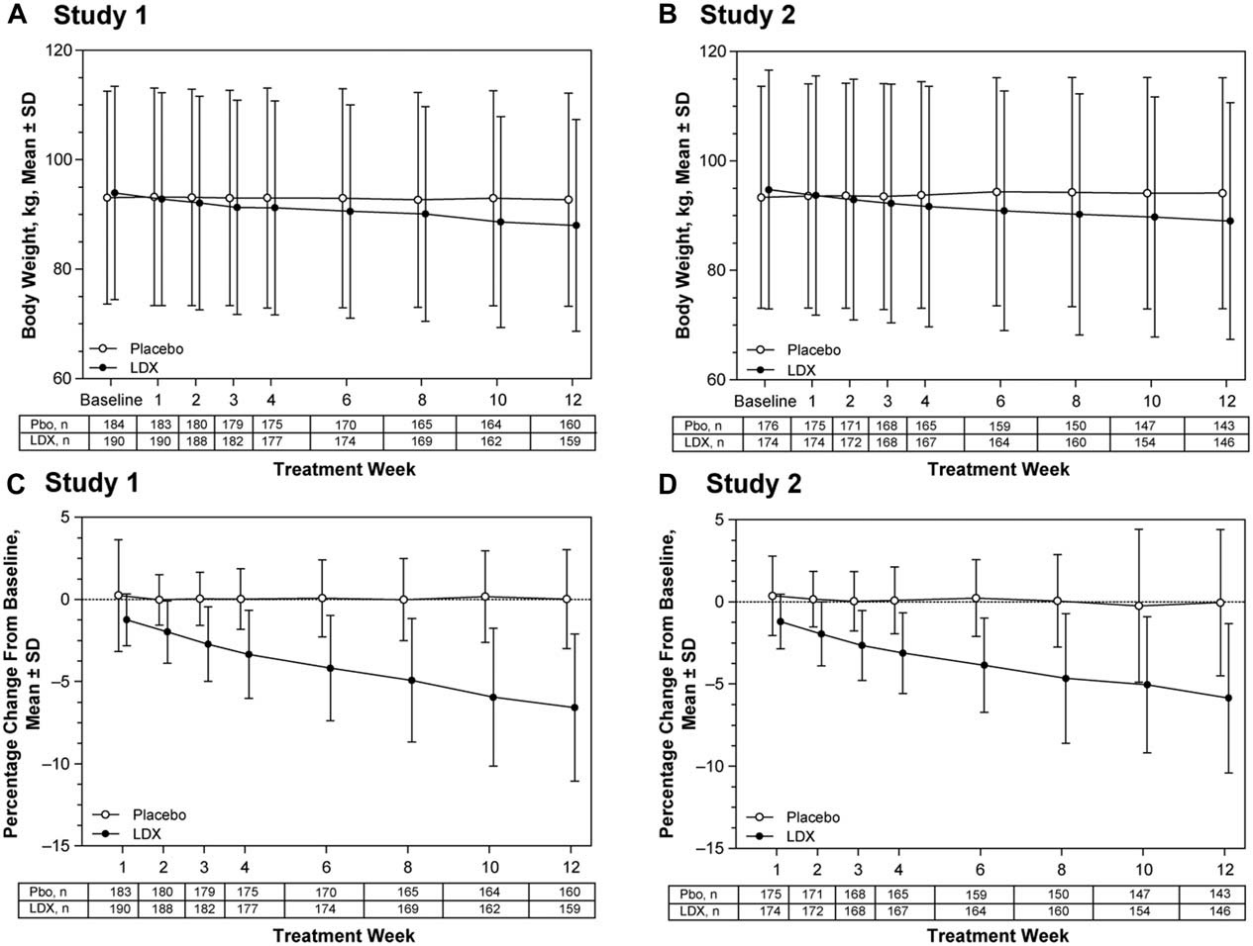
Reductions in body weight over time, full analysis set. Mean ± *SD* body weight in kg (A: Study 1; B: Study 2) and mean ± *SD* percentage changes in body weight from baseline (C: Study 1; D: Study 2). Abbreviations: ET = early termination; LDX = lisdexamfetamine; Pbo=placebo

Mean ± *SD* body weight decreased with LDX but not placebo over the course of both studies (Figure 4A,B), resulting in larger mean ± *SD* percentage decreases in body weight from baseline with LDX than placebo (Figure 4C,D). In both studies, the LS mean (95% CI) treatment differences for the percentage body weight change from baseline favored LDX over placebo from Week 1 through Week 10 (all nominal *p* values < .001; all ES ≥ .56) and at Week 12 (both *p* values < .001; both ES ≥ 1.22) (Supporting Information Table 2).

#### 3.2.3 Y‐BOCS‐BE total and domain scores

**Figure 5:**
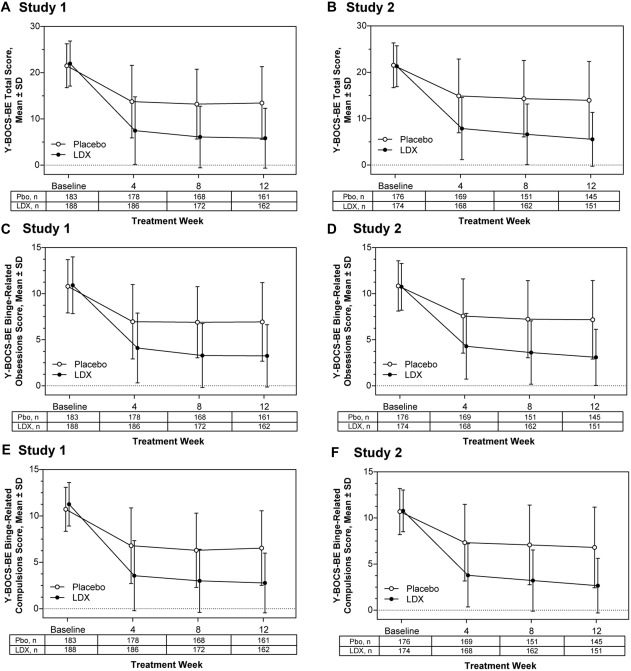
Y‐BOCS‐BE total and domain scores over time, full analysis set. Mean ± *SD* Y‐BOCS‐BE total scores (A: Study 1; B: Study 2), mean ± *SD* Y‐BOCS‐BE binge‐related obsessions domain scores (C: Study 1; D: Study 2), and mean ± *SD* binge‐related compulsions domain scores (E: Study 1; F: Study 2). Abbreviations: LDX=lisdexamfetamine; Pbo=placebo; Y‐BOCS‐BE=Yale‐Brown Obsessive Compulsive Scale modified for Binge Eating

Mean ± *SD* Y‐BOCS‐BE total scores (Figure 5A,B) and domain scores (Figure 5C–F) decreased (i.e., improved) from baseline with placebo and LDX during both studies at each of the three postbaseline assessment time points. For Y‐BOCS‐BE total score changes from baseline (Supporting Information Table 2), LS mean (95% CI) treatment differences favored LDX at Week 4 and Week 8 (all nominal *p* values < .001; all ES ≥ .87) and at Week 12 (both *p* values < .001; both ES ≥ 1.03) in both studies. For the binge‐related obsessions and binge‐related compulsions domain scores, LS mean (95% CI) treatment differences also favored LDX over placebo at Weeks 4, 8, and 12 (all nominal *p* values < .001; all ES ≥ .78; Supporting Information Table 2) in both studies.

## 4 DISCUSSION

The key findings of this report are that the treatment effects of LDX versus placebo were observed as early as the first week of treatment across multiple endpoints (binge eating days/week, binge eating episodes/week, 1‐week binge eating response, dichotomized improvement on the CGI‐I, and percentage change in body weight from baseline) and at Week 4 on Y‐BOCS‐BE total and domain scores (the first time point assessed). Furthermore, improvements were maintained for the duration of the 12‐week studies. These results support the relatively rapid efficacy of LDX in reducing both binge eating behavior and binge eating‐related psychopathology in two studies (McElroy et al., 2015a) in which 12 weeks of dose‐optimized LDX was shown to produce significantly greater improvement than placebo at the end of the study on several of these same measures. Consistent with the results of the Phase 2 study (McElroy et al., 2015b), this post hoc analysis revealed a possible treatment effect at Week 1, when 30 mg LDX was being taken by study participants. This finding is difficult to explain because 30 mg LDX was not statistically superior to placebo in reducing log‐transformed binge eating days/week at the Week 11 (the primary endpoint) in the Phase 2 study (McElroy et al., 2015b). Although 30 mg LDX is a recommended starting titration dose for the treatment of BED (Vyvanse^®^, 2015), it was not studied as a target treatment dose based largely on the Phase 3 pivotal studies in which 30 mg LDX was used only as an initial titration dose and not studied as a target dose (McElroy et al., 2015a). Additional research with a study design focused on this research question would be needed to draw firmer conclusions.

Although medications other than LDX have been investigated for potential use as BED pharmacotherapy in double‐blind, placebo‐controlled trials, LDX is the only medication currently approved in the United States for the treatment of adults with moderate to severe BED (Vyvanse^®^, 2015). To our knowledge, this is the first publication describing time course analyses of treatment effects by week for the efficacy of a pharmacotherapy in individuals with BED. Based on available data from short‐term studies for other potential BED therapies, numerically greater reductions in binge eating frequency and body weight for active treatment versus placebo are generally observed within 1–4 weeks of starting treatment (Appolinario et al., 2003; Guerdjikova et al., 2012; Hudson et al., 1998; McElroy et al., 2003, 2000, 2007; Wilfley et al., 2008). In one study that reported statistical analysis of treatment effects prior to the end of the study, significantly greater reductions in binge eating days/week and weight for sibutramine versus placebo were reported at treatment Weeks 2 and 4, respectively (Appolinario et al., 2003).

The study has several limitations. These time course analyses were not prespecified, were not included in the hierarchical testing strategy, and did not account for multiple comparisons. Therefore, all findings related to the time course of effects of LDX are nominal (unadjusted for multiplicity and descriptive in nature). In addition, study participants were mainly women, mainly white, and predominantly met criteria for obesity. Study participants with current comorbid psychiatric disorders that were controlled with prohibited medications or uncontrolled and associated with significant symptoms were also excluded, as were those with histories of psychosis, mania/hypomania, and ADHD (those with mild mood or anxiety symptoms that did not meet diagnostic criteria or require treatment could be included), which may limit the generalizability of the current findings to a more heterogeneous clinical population. Lastly, it has been reported that in a majority of individuals with bulimia nervosa (combined data from two studies; *n* = 785) eventual nonresponse to fluoxetine at the end of 7–8 weeks of fluoxetine treatment is unlikely if reductions in binge eating or vomiting of at least 60% are not observed after 3 weeks of fluoxetine treatment (Sysko, Sha, Wang, Duan, & Walsh, 2010). It would also be of interest to assess the relationship between early treatment response to LDX and long‐term outcomes in individuals with BED. However, these analyses were not conducted so the degree to which short‐term response to LDX predicts long‐term outcomes cannot be determined.

In conclusion, LDX appeared to be associated with improvement in efficacy‐related endpoints in adults with protocol‐defined moderate to severe BED after 1 week of treatment (after Week 4 [the first assessment] for the Y‐BOCS‐BE) and these improvements were maintained for the course of the 12‐week treatment period. These results suggest that LDX reduces both binge eating behavior and binge eating‐related psychopathology soon after treatment is initiated.

## Supporting information

Additional Supporting Information may be found online in the supporting information tab for this article.

Supporting Information Table 1.

Supporting Information Table 2.
