# Supplementary material for: Time course of the effects of lisdexamfetamine dimesylate in two phase 3, randomized, double‐blind, placebo‐controlled trials in adults with binge‐eating disorder
Source: Int J Eat Disord. 2017 May 8;50(8):884–92. doi: 10.1002/eat.22722 (PMC5573905; doi:10.1002/eat.22722)
Supplement: Supplementary file 1 — Supporting Information Table 1. [file EAT-50-884-s001.docx]

**Supplemental Table 1. Change From Baseline Treatment Differences^a^ for Binge Eating Days/Week and Binge Eating Episodes/Week, Full Analysis Set**

|  | |  | | **Study 1** | | |  |  | | **Study 2** | |  |
| --- | --- | --- | --- | --- | --- | --- | --- | --- | --- | --- | --- | --- |
| **Week** | **N**  **Pbo, LDX** | | **LS Mean**  **(95% CI)^a^** | | **t-statistic (DF);  *P* Value ^b^** | **ES^c^** | | **N**  **Pbo, LDX** | **LS Mean**  **(95% CI)^a^** | | **t-statistic (DF);  *P* Value^b^** | **ES^c^** |
| Binge eating days/week | | | | | |  | |  | | | |  |
| 1 | 184,190 | | –1.21 (–1.58, –0.85) | | –6.47 (371); *P*<0.001 | 0.67 | | 176,174 | –1.00 (–1.37, –0.63) | | –5.34 (347); *P*<0.001 | 0.57 |
| 2 | 180,186 | | –1.55 (–1.93, –1.17) | | –8.04 (366); *P*<0.001 | 0.84 | | 171,173 | –1.33 (–1.72, –0.94) | | –6.77 (345); *P*<0.001 | 0.73 |
| 3 | 179,182 | | –1.67 (–2.04, –1.30) | | –8.96 (362); *P*<0.001 | 0.94 | | 167,169 | –1.46 (–1.85, –1.07) | | –7.35 (344); *P*<0.001 | 0.80 |
| 4 | 173,177 | | –1.48 (–1.84, –1.11) | | –7.95 (362); *P*<0.001 | 0.84 | | 164,167 | –1.58 (–1.99, –1.18) | | –7.72 (340); *P*<0.001 | 0.84 |
| 5–6 | 169,173 | | –1.65 (–1.98, –1.32) | | –9.74 (361); *P*<0.001 | 1.03 | | 158,162 | –1.51 (–1.90, –1.13) | | –7.73 (335); *P*<0.001 | 0.85 |
| 7–8 | 165,167 | | –1.38 (–1.71, –1.05) | | –8.18 (361); *P*<0.001 | 0.87 | | 149,158 | –1.53 (–1.90, –1.15) | | –8.05 (336); *P*<0.001 | 0.89 |
| 9–10 | 164,162 | | –1.41 (–1.75, –1.06) | | –8.02 (361); *P*<0.001 | 0.86 | | 146,153 | –1.57 (–1.95, –1.20) | | –8.20 (334); *P*<0.001 | 0.91 |
| 11–12^d^ | 160,158 | | –1.35 (–1.70, –1.01) | | –7.67 (350); *P*<0.001 | 0.83 | | 142,146 | –1.66 (–2.04, –1.28) | | –8.60 (331); *P*<0.001 | 0.97 |
| Binge eating episodes/week | | | | | |  | |  | | | |  |
| 1 | 184,190 | | –1.73 (–2.28, –1.18) | | –6.15 (371); *P*<0.001 | 0.64 | | 176,174 | –1.65 (–2.23, –1.07) | | –5.60 (347); *P*<0.001 | 0.60 |
| 2 | 180,186 | | –2.14 (–2.72, –1.57) | | –7.31 (366); *P*<0.001 | 0.76 | | 171,173 | –2.00 (–2.59, –1.40) | | –6.60 (348); *P*<0.001 | 0.71 |
| 3 | 179,182 | | –2.17 (–2.72, –1.62) | | –7.71 (362); *P*<0.001 | 0.81 | | 167,169 | –2.02 (–2.59, –1.45) | | –6.96 (344); *P*<0.001 | 0.75 |
| 4 | 173,177 | | –1.95 (–2.49, –1.42) | | –7.15 (366); *P*<0.001 | 0.76 | | 164,167 | –2.25 (–2.82, –1.67) | | –7.72 (341); *P*<0.001 | 0.84 |
| 5–6 | 169,173 | | –2.13 (–2.62, –1.64) | | –8.58 (365); *P*<0.001 | 0.91 | | 158,162 | –2.12 (–2.67, –1.57) | | –7.54 (338); *P*<0.001 | 0.82 |
| 7–8 | 165,167 | | –1.78 (–2.25, –1.31) | | –7.50 (365); *P*<0.001 | 0.80 | | 149,158 | –2.07 (–2.60, –1.53) | | –7.58 (336); *P*<0.001 | 0.83 |
| 9–10 | 164,162 | | –1.82 (–2.30, –1.34) | | –7.46 (367); *P*<0.001 | 0.80 | | 146,153 | –2.20 (–2.76, –1.64) | | –7.75 (335); *P*<0.001 | 0.85 |
| 11–12 | 160,158 | | –1.77 (–2.24, –1.30) | | –7.40 (357); *P*<0.001 | 0.79 | | 142,146 | –2.23 (–2.77, –1.69) | | –8.14 (333); *P*<0.001 | 0.91 |

DF=degrees of freedom; ES=effect size; LDX=lisdexamfetamine; LS=least squares; Pbo=placebo.

^a^Treatment differences calculated as lisdexamfetamine – placebo; negative values favor lisdexamfetamine over placebo.

^b^Based on mixed-effects models for repeated measures analysis over all postbaseline visits using an unstructured covariance matrix, with treatment, visit, and the treatment × visit interaction included as factors and baseline score as a covariate and its interaction with visit also in the model. All reported *P* values are nominal, except for binge eating days/week at weeks 11–12 which was the prespecified primary efficacy endpoint. Degrees of freedom were calculated using the Kenward-Roger approximation method.

^c^Effect size is based on the estimated standard deviation from the unstructured covariane matrix.

**^d^**Prespecified primary endpoint (data previously reported^2^).
