# Supplementary material for: Time course of the effects of lisdexamfetamine dimesylate in two phase 3, randomized, double‐blind, placebo‐controlled trials in adults with binge‐eating disorder
Source: Int J Eat Disord. 2017 May 8;50(8):884–92. doi: 10.1002/eat.22722 (PMC5573905; doi:10.1002/eat.22722)
Supplement: Supplementary file 2 — Supporting Information Table 2. [file EAT-50-884-s002.docx]

**Supplemental Table 2. Treatment Differences^a^ in Percentage Weight Change and Y-BOCS-BE Scores From Baseline, Full Analysis Set**

|  | | **Study 1** | | | |  | **Study 2** | | | |  |
| --- | --- | --- | --- | --- | --- | --- | --- | --- | --- | --- | --- |
| **Week** | **N**  **Pbo, LDX** | | **LS mean**  **(95% CI)^a^** | **t-statistic (DF);**  ***P* Value^b^** | **ES^c^** | | **N**  **Pbo, LDX** | **LS mean**  **(95% CI)^a^** | **t-statistic (DF);**  ***P* Value^b^** | **ES^c^** | |
| Percentage body weight change from baseline | | | | |  | |  | | |  | |
| 1 | | 183,190 | –1.48 (–2.02, –0.95) | –5.44 (371); *P*<0.001 | 0.56 | | 175,174 | –1.56 (–2.00, –1.13) | –7.03 (346); *P*<0.001 | 0.75 | |
| 2 | | 180,188 | –1.95 (–2.30, –1.60) | –10.90 (368); *P*<0.001 | 1.14 | | 171,172 | –2.11 (–2.50, –1.73) | –10.79 (344); *P*<0.001 | 1.16 | |
| 3 | | 179,182 | –2.77 (–3.17, –2.36) | –13.47 (366); *P*<0.001 | 1.41 | | 168,168 | –2.73 (–3.15, –2.31) | –12.88 (343); *P*<0.001 | 1.40 | |
| 4 | | 175,177 | –3.37 (–3.84, –2.90) | –14.09 (367); *P*<0.001 | 1.48 | | 165,167 | –3.19 (–3.67, –2.72) | –13.20 (343); *P*<0.001 | 1.43 | |
| 6 | | 170,174 | –4.27 (–4.84, –3.69) | –14.58 (366); *P*<0.001 | 1.54 | | 159,164 | –4.01 (–4.57, –3.45) | –14.15 (342); *P*<0.001 | 1.54 | |
| 8 | | 165,169 | –4.96 (–5.63, –4.29) | –14.60 (359); *P*<0.001 | 1.55 | | 150,160 | –4.58 (–5.32, –3.84) | –12.21 (340); *P*<0.001 | 1.35 | |
| 10 | | 164,162 | –6.02 (–6.77, –5.26) | –15.68 (354); *P*<0.001 | 1.67 | | 147,154 | –4.56 (–5.52, –3.61) | –9.40 (335); *P*<0.001 | 1.05 | |
| 12^d^ | | 160,159 | –6.35 (–7.17, –5.54) | –15.31 (350); *P*<0.001 | 1.64 | | 143,146 | –5.41 (–6.39, –4.44) | –10.89 (335); *P*<0.001 | 1.22 | |
| Y-BOCS-BE total score | | | | |  | |  | | |  | |
| 4 | 177,184 | | –6.53 (–8.09, –4.98) | –8.27 (358); *P*<0.001 | 0.87 | | 169,168 | –7.12 (–8.65, –5.60) | –9.19 (335); *P*<0.001 | 1.00 | |
| 8 | 168,170 | | –6.93 (–8.43, –5.44) | –9.13 (350); *P*<0.001 | 0.97 | | 151,162 | –7.56 (–9.15, –5.97) | –9.36 (323); *P*<0.001 | 1.04 | |
| 12^d^ | 161,160 | | –7.40 (–8.93, –5.88) | –9.55 (344); *P*<0.001 | 1.03 | | 145,151 | –7.94 (–9.51, –6.36) | –9.90 (312); *P*<0.001 | 1.11 | |
| Y-BOCS-BE binge-related obsessions domain score | | | | |  | |  | | |  | |
| 4 | 177,184 | | –3.02 (–3.82, –2.23) | –7.47 (357); *P*<0.001 | 0.78 | | 169,168 | –3.40 (–4.19, –2.60) | –8.43 (335); *P*<0.001 | 0.92 | |
| 8 | 168,170 | | –3.61 (–4.38, –2.84) | –9.26 (351); *P*<0.001 | 0.99 | | 151,162 | –3.64 (–4.46, –2.82) | –8.72 (321); *P*<0.001 | 0.97 | |
| 12 | 161,160 | | –3.66 (–4.47, –2.85) | –8.86 (346); *P*<0.001 | 0.96 | | 145,151 | –3.88 (–4.69, –3.06) | –9.33 (308); *P*<0.001 | 1.05 | |
| Y-BOCS-BE binge-related compulsions domain score | | | | |  | |  | | |  | |
| 4 | 177,184 | | –3.48 (–4.29, –2.66) | –8.40 (357); *P*<0.001 | 0.88 | | 169,168 | –3.73 (–4.53, –2.94) | –9.24 (335); *P*<0.001 | 1.01 | |
| 8 | 168, 170 | | –3.33 (–4.11, –2.54) | –8.31 (347); *P*<0.001 | 0.89 | | 151,162 | –3.94 (–4.77 –3.12) | –9.41 (324); *P*<0.001 | 1.05 | |
| 12 | 161, 160 | | –3.76 (–4.54, –2.98) | –9.48 (339); *P*<0.001 | 1.03 | | 145,151 | –4.10 (–4.92, –3.28) | –9.88 (313); *P*<0.001 | 1.12 | |

DF=degrees of freedom; ES=effect size; LDX=lisdexamfetamine; LS=least squares; Pbo=placebo; Y-BOCS-BE=Yale-Brown Obsessive Compulsive Scale modified for Binge Eating.

^a^Treatment differences calculated as lisdexamfetamine – placebo; negative values favor lisdexamfetamine over placebo.

^b^Based on mixed-effects models for repeated measures analysis over all postbaseline visits using an unstructured covariance matrix, with treatment, visit, and the treatment × visit interaction included as factors and baseline score as a covariate. All reported *P* values are nominal except for percentage body weight change and Y-BOCS-BE total score change at week 12, which were included in the prespecified hierarchical testing strategy. Degrees of freedom were calculated using the Kenward-Roger approximation method.

**^c^**Effect size is based on the estimated standard deviation from the unstructured covariane matrix.

**^d^**Prespecified key secondary endpoint (data previously reported^2^).
